# DOA Finding with Support Vector Regression Based Forward–Backward Linear Prediction

**DOI:** 10.3390/s17061225

**Published:** 2017-05-27

**Authors:** Jingjing Pan, Yide Wang, Cédric Le Bastard, Tianzhen Wang

**Affiliations:** 1 Institut d’Electronique et Télécommunications de Rennes, UMR CNRS 6164, Polytech Nantes, Rue Christian Pauc, BP 50609, 44306 Nantes CEDEX 3, France; jingjing.pan1@etu.univ-nantes.fr (J.P.); cedric.lebastard@cerema.fr (C.L.B.); 2 Cerema, 49136 Les Ponts de Cé, France; 3 Logistics Engineering College, Shanghai Maritime University, Shanghai 201306, China; tzwang@shmtu.edu.cn

**Keywords:** direction-of-arrival (DOA), support vector regression (SVR), forward–backward linear prediction (FBLP), coherent signals, low snapshots

## Abstract

Direction-of-arrival (DOA) estimation has drawn considerable attention in array signal processing, particularly with coherent signals and a limited number of snapshots. Forward–backward linear prediction (FBLP) is able to directly deal with coherent signals. Support vector regression (SVR) is robust with small samples. This paper proposes the combination of the advantages of FBLP and SVR in the estimation of DOAs of coherent incoming signals with low snapshots. The performance of the proposed method is validated with numerical simulations in coherent scenarios, in terms of different angle separations, numbers of snapshots, and signal-to-noise ratios (SNRs). Simulation results show the effectiveness of the proposed method.

## 1. Introduction

Direction-of-arrival (DOA) estimation is of great importance in array signal processing [1,2]. A considerable number of techniques have been developed for the determination of the DOAs of incoming signals. Fourier-based methods belong to the traditional fast processing methods [1], but the resolution capability of the Fourier-based methods is limited by the physical size of the array (aperture). Subspace methods such as multiple signal classification (MUSIC) [3] and estimation of signal parameters via rational invariance technique (ESPRIT) [4] are popular because of their asymptotic infinite resolution and unbiased estimation performance. Apart from the successful applications in DOA finding, they are also very attractive in time-reversal imaging [5,6,7]. However, subspace methods require the eigendecomposition of the covariance matrix of the received signal to get the noise and signal subspaces, which adds to the computational burden. Besides, subspace methods cannot be applied directly in coherent scenarios [8]. Additional decorrelation techniques (e.g., spatial smoothing, SS) are usually necessary. SS was originally proposed for DOA estimation with coherent signals in [9], which was only a single-direction SS technique. Since then, various kinds of improvements of this technique have been developed. A forward–backward (FB) processing technique was introduced in [10] to improve the performance of the single-direction one. The work in [11] aimed at improving the computational efficiency of FB. In [12], the cross correlation in each sub-array was employed together with auto-correlation in SS processing. The authors in [13] focused on the correlated noise in coherent scenarios using FB. DOA finding of multi-group coherent signals was considered in [14]. Linear prediction (LP) is commonly used in time series problems, and can also be used in array signal processing [15]. LP makes use of multiple overlapping observation sequences for the prediction of unknown ones. Similar to SS, it can directly deal with coherent signals [9,15]. It does not require eigendecomposition of the covariance matrix of the received signals. Besides, the family of LP methods has a higher resolution than the conventional Fourier-based approaches. However, LP fails to work when the number of snapshots is small [15,16].

The theory of LP is based on finding the weights which minimize the prediction error. Support vector regression (SVR) is a good sparse machine learning method capable of dealing with small samples [17]. Originally, the formulation of SVR was applied within the real domain, and later it was extended to complex data [18,19,20]. In the literature, SVR has many applications for signal processing problems, which can be classified into two approaches. One uses the training and testing pattern of SVR to approximate the mapping between the variables to be estimated and known features. The features are usually elements in the covariance matrix of the received signals. Details can be found in the estimation of DOA [21] and time delay [22]. The other approach combines the theory of SVR with classical signal processing methods. In [19], an SVR-based beamforming method was proposed to control the level of beam sidelobes. Similarly, the authors in [20] formulated SVR with Capon and then MUSIC in the estimation of the DOAs of incoming waves. However, [20] depended on an additional SS method or a recursive approach in coherent scenarios. The work in [20] also required the repetition of the SVR procedure along the DOA spectrum, which needs more computational time. Besides, there are combinations of SVR with linear signal processing methods such as auto-regressive model (AR) [18] and auto-regressive moving average (ARMA) [23] for frequency estimation and system identification problems. As described in [24], the principle of AR is closely related to the forward linear prediction (FLP). However, forward–backward linear prediction (FBLP) offers better performance than the one-directional prediction methods (FLP and backward linear prediction, BLP) [25]. Moreover, there is no explicit work about SVR-based LP models with coherent signals. Therefore, we propose to combine SVR with FBLP in the estimation of the DOAs of coherent incoming signals with a small number of snapshots.

The rest of this paper is organized as follows. Section 2 presents the signal model. Section 3 describes the framework of FBLP and the proposed SVR-based FBLP method. Section 4 gives some simulation results to show the effectiveness of the proposed method. Section 5 draws the conclusion.

Notations: ${\left(.\right)}^{T}$, ${\left(.\right)}^{*}$, and ${\left(.\right)}^{H}$ denote the transpose, conjugate, and transpose conjugate operations, respectively. R and C denote real and complex numbers, respectively. Vectors and matrices appear in boldface lowercase letters and boldface capital letters, respectively. Re(z) and Im(z) denote the real and imaginary parts of z, respectively.

## 2. Signal Model

Consider a uniform linear array (ULA) with *M* isotropic antenna elements. There are *K* far-field narrow band incoming signals impinging on the antennas, which are corrupted by an additive Gaussian white noise (AGWN). If the number of snapshots is *L*, the output of antenna *m* can be expressed as [15]:

(1) $$x_{m}={\left[x_{m}\left(1\right),\;x_{m}\left(2\right),\;\cdots ,\;x_{m}\left(L\right)\right]}^{T}$$

with

(2) $$x_{m}\left(t\right)=\sum_{k=1}^{K}s_{k}\left(t\right)e^{j\left(m-1\right)2\pi \frac{d}{\lambda }sin\left(\theta_{k}\right)}+n_{m}\left(t\right)$$

where $s_{k}\left(t\right)$ denotes the *k*th incoming signal received at the first antenna; $\theta_{k}$ is the corresponding DOA of the *k*th signal (with respect to the normal line of the array); $n_{m}\left(t\right)$ is the AGWN at the *m*th antenna; *d* denotes the distance between two adjacent sensors; and λ is the wavelength of the incoming signals.

## 3. Methodology

### 3.1. Forward–Backward Linear Prediction

LP methods estimate unknown values with a weighted sum of the known observations by minimizing the mean square prediction error. FBLP makes use of the observations from both forward and backward sequences in the estimation. When the order of the prediction filter is *P*, the prediction equation in matrix form can be expressed as follows [15,26]:

(3) $$\left[\begin{array}{cccc} x_{P} & x_{P-1} & \ldots & x_{1}\\ \vdots & \vdots & & \vdots \\ x_{M-1} & x_{M-2} & \cdots & x_{M-P}\\ \cdots \cdots & \cdots \cdots & \cdots \cdots & \cdots \cdots \\ x_{2}^{*} & x_{3}^{*} & \cdots & x_{P+1}^{*}\\ \vdots & \vdots & \vdots & \\ x_{M-P+1}^{*} & x_{M-P+2}^{*} & \cdots & x_{M}^{*} \end{array}\right]\left[\begin{array}{c} \omega_{1}\\ \omega_{2}\\ \vdots \\ \omega_{P} \end{array}\right]=\left[\begin{array}{c} x_{P+1}\\ \vdots \\ x_{M}\\ \cdots \cdots \\ x_{1}^{*}\\ \vdots \\ x_{M-P}^{*} \end{array}\right]$$

We can observe that FLP and BLP can be expressed with the first and second halves (above and below the dotted line) of Equation (3), respectively. In a more compact way, Equation (3) can be rewritten as:

(4) Xω=y

where $y\in C^{N_{T}\times 1}$, $X\in C^{N_{T}\times P}$ and $\omega \in C^{P\times 1}$, $N_{T}=2\left(M-P\right)L$.

The weight coefficient vector ω can be estimated by the following equation:

(5) $$\omega =R^{-1}r$$

where $R=X^{H}X/L$ and $r=X^{H}y/L$. The reverse of R requires the inequality: $N_{T}⩾P$ so that R is a non-singular matrix. Hence we have the constraint for the number of snapshots *L* as L⩾P/[2(M−P)].

According to [26], the order of the prediction filter *P* should satisfy: K⩽P⩽M−K/2.

With the estimated weight vector ω, the power spectrum density (PSD) of FBLP can be expressed as:

(6) $$P\left(\theta \right)=\frac{1}{|a^{H}\left(\theta \right)\left[\begin{array}{c} 1\\ -\omega \end{array}\right]{|}^{2}}$$

with $a\left(\theta \right)={\left[1,e^{j2\pi \frac{d}{\lambda }sin\left(\theta \right)},\cdots ,e^{j2\pi P\frac{d}{\lambda }sin\left(\theta \right)}\right]}^{T}$, which is the steering vector.

### 3.2. Proposed Method: FBLP-SVR

The key issue in LP methods is to find the weight vector ω. However, in scenarios where the observation is insufficient (i.e., the number of snapshots is too small), LP methods might be unstable (even unsuccessful). Therefore, we propose to use the principle of SVR to deal with the situations of small samples. Inspired by [19], we propose a combined method of FBLP and SVR, which is called FBLP-SVR in the following.

According to [19], Equation (4) can be rewritten in terms of real and imaginary parts as:

(7) $$\tilde{y}=\bar{X}\tilde{\omega }$$

where ${\tilde{\omega }}^{T}=\left[Re\left(\omega^{T}\right)\;Im\left(\omega^{T}\right)\right]$, $\bar{X}=\left[\begin{array}{cc} Re(X) & -Im(X)\\ Im(X) & Re(X) \end{array}\right]$ and ${\tilde{y}}^{T}=\left[Re\left(y^{T}\right)\;Im\left(y^{T}\right)\right]$.

It is worth noting that Equation (7) can be viewed as a typical form of SVR in real domain. The transformed vector $\tilde{\omega }$ belongs to $R^{2P\times 1}$, while $\tilde{y}\in R^{2N_{T}\times 1}$ and $\bar{X}\in R^{2N_{T}\times 2P}$. In regression problems, the objective of SVR is to build a hyper-plane to fit the data within a deviation less than a given value ϵ. We adopt the ϵ-intensive loss function here [17]. The optimization problem is to minimize the regression error both structurally and empirically:

(8) $$\min_{\tilde{\omega },\xi ,\tilde{\xi }}\;L_{p}\left(\tilde{\omega },\xi ,\tilde{\xi }\right)=\min_{\tilde{\omega },\xi ,\tilde{\xi }}\;\left[\frac{1}{2}{∥\tilde{\omega }∥}^{2}+C\sum_{i=1}^{2N_{T}}\left(\xi_{i}+{\tilde{\xi }}_{i}\right)\right]$$

$$s.t.\;\left\{\begin{array}{c} {\tilde{y}}_{i}-{\bar{x}}_{i}^{T}\tilde{\omega }⩽\epsilon +\xi_{i},i=1,\cdots ,2N_{T}\\ -{\tilde{y}}_{i}+{\bar{x}}_{i}^{T}\tilde{\omega }⩽\epsilon +{\tilde{\xi }}_{i},i=1,\cdots ,2N_{T}\\ \xi_{i},{\tilde{\xi }}_{i}⩾0,i=1,\cdots ,2N_{T} \end{array}\right.$$

where ${\bar{x}}_{i}$ and ${\tilde{y}}_{i}$ are the *i*th column of ${\bar{X}}^{T}$ and ${\tilde{y}}^{T}$, respectively; $\xi_{i}$ and ${\tilde{\xi }}_{i}$ are the corresponding slack variables to compensate for empirical errors; *C* controls the trade-off between the model complexity and empirical error.

The solution of Equation (8) is to construct a Lagrange function from the objective function (also called primal objective function) and the constraints by introducing a dual set of variables. The constructed Lagrange function has a saddle point that minimizes over the primal variables and maximizes over the dual variables [17]. Therefore, we deduce the following primal–dual objective function:

(9) $$\begin{array}{cc} L_{pd}= & \frac{1}{2}{∥\tilde{\omega }∥}^{2}+C\sum_{i=1}^{2N_{T}}\left(\xi_{i}+{\tilde{\xi }}_{i}\right)-\sum_{i=1}^{2N_{T}}\left(\lambda_{i}\xi_{i}+{\tilde{\lambda }}_{i}{\tilde{\xi }}_{i}\right)\\ & \;+\sum_{i=1}^{2N_{T}}\alpha_{i}\left({\tilde{y}}_{i}-{\bar{x}}_{i}^{T}\tilde{\omega }-\epsilon -\xi_{i}\right)\\ & \;+\sum_{i=1}^{2N_{T}}{\tilde{\alpha }}_{i}\left(-{\tilde{y}}_{i}+{\bar{x}}_{i}^{T}\tilde{\omega }-\epsilon -{\tilde{\xi }}_{i}\right) \end{array}$$

where $\alpha_{i}$, ${\tilde{\alpha }}_{i}$, $\lambda_{i}$, and ${\tilde{\lambda }}_{i}$ are Lagrange multipliers.

All the partial derivatives of $L_{pd}$ with respect to the primal variables ($\tilde{\omega }$, $\xi_{i}$, and ${\tilde{\xi }}_{i}$) should be equal to zero at the saddle point:

(10) $$\left\{\begin{array}{c} \frac{\partial L_{pd}}{\partial \tilde{\omega }}=\tilde{\omega }-\sum_{i=1}^{2N_{T}}\left(\alpha_{i}-{\tilde{\alpha }}_{i}\right){\bar{x}}_{i}=0\\ \frac{\partial L_{pd}}{\partial \xi_{i}}=C-\alpha_{i}-\lambda_{i}=0,\;i=1,\cdots ,2N_{T}\\ \frac{\partial L_{pd}}{\partial {\tilde{\xi }}_{i}}=C-{\tilde{\alpha }}_{i}-{\tilde{\lambda }}_{i}=0,\;i=1,\cdots ,2N_{T} \end{array}\right.$$

The weight vector $\tilde{\omega }$ is reformulated by means of the Lagrange multipliers: $\tilde{\omega }=\sum_{i=1}^{2N_{T}}\left(\alpha_{i}-{\tilde{\alpha }}_{i}\right){\bar{x}}_{i}$. Substituting Equation (10) into Equation (9) yields the dual optimization problem:

(11) $$\begin{array}{c} \max_{\alpha ,\tilde{\alpha }}L_{d}\left(\alpha ,\tilde{\alpha }\right)=\max_{\alpha ,\tilde{\alpha }}\;\left[-\frac{1}{2}{\left(\alpha -\tilde{\alpha }\right)}^{T}\left(\bar{X}\;{\bar{X}}^{T}+\gamma I\right)\left(\alpha -\tilde{\alpha }\right)-\epsilon 1^{T}\left(\alpha +\tilde{\alpha }\right)+{\tilde{y}}^{T}\left(\alpha -\tilde{\alpha }\right)\right] \end{array}$$

$$s.t.\;\;0⩽\alpha_{i},{\tilde{\alpha }}_{i}⩽C,\;\;i=1,\cdots ,2N_{T}$$

where $\alpha ={\left[\alpha_{1},\alpha_{2},\cdots ,\alpha_{2N_{T}}\right]}^{T}$ and $\tilde{\alpha }={\left[{\tilde{\alpha }}_{1},{\tilde{\alpha }}_{2},\cdots ,{\tilde{\alpha }}_{2N_{T}}\right]}^{T}$; 1 is an all-one column vector with $2N_{T}$ elements.

In Equation (11), α and $\tilde{\alpha }$ are the coefficients vectors maximizing the quadratic objective function, which can be found using any quadratic programming (QP) solvers [20]. A small identity term γI is added in the dual objective function in case of ill-conditional matrix of $\bar{X}\;{\bar{X}}^{T}$ in Equation (11) [18]. The QP techniques are computationally demanding. Nevertheless, the number of samples in the considered situations being small, the increase of the computational burden is not significant, which will be shown qualitatively in the simulation part.

Finally, the weight vector $\tilde{\omega }$ is obtained. We can rewrite it back into the complex domain as $\omega_{i}={\tilde{\omega }}_{i}+j{\tilde{\omega }}_{i+P}$ for i=1,⋯,P and then find the DOAs by searching the peak positions of the power spectrum density defined in Equation (6).

## 4. Simulation Results

In this section, the performance of the proposed method is evaluated with four simulations. We assume a ULA with ten isotropic sensors (i.e., M=10). The distance between two adjacent sensors is half the wavelength of incoming signals, d=λ/2.

The research results in [15] show that the decorrelation ability of LP methods is at the expense of the real effective array aperture. In order to maintain an effective array aperture, we chose *P* = 9. Therefore, the number of snapshots *L* in the standard FBLP should be greater than five in order to make the covariance matrix invertible. The SVR parameters used in all the simulations are ϵ=0.1, C=0.1, and $\gamma =10^{-6}$, as in [20].

### 4.1. Performance with Power Spectrum Density

**Figure 1:**
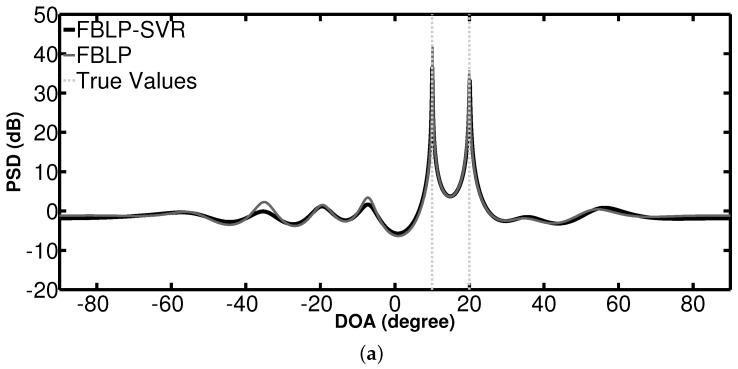

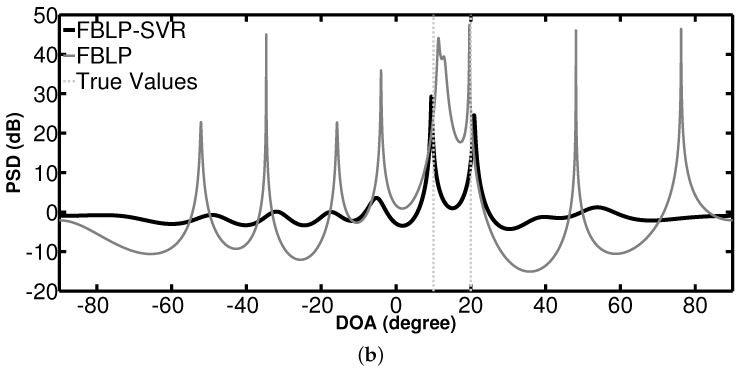
Power spectrum density (PSD) of combined forward–backward linear prediction and support vector regression (FBLP-SVR), and FBLP with 10 antennas and 2 coherent signals coming from $\theta_{1}=10^{\circ }$, $\theta_{2}=20^{\circ }$. (**a**) number of snapshots = 100; (**b**) number of snapshots = 5.

**Figure 2:**
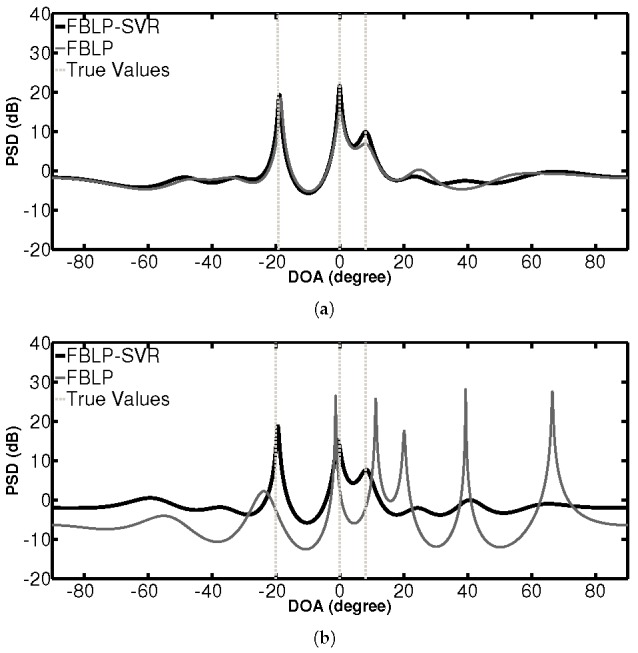
PSD of FBLP-SVR and FBLP with 10 antennas and 3 coherent signals coming from $\theta_{1}=-20^{\circ }$, $\theta_{2}=0^{\circ }$, $\theta_{3}=8^{\circ }$. (**a**) number of snapshots = 100; (**b**) number of snapshots = 5.

In the first simulation, we examine the PSD of the standard FBLP and the proposed FBLP-SVR not only with two sources, but also with three sources. In the two-source case, the signals come from $\theta_{1}=10^{\circ }$, $\theta_{2}=20^{\circ }$. When there are three sources, the signals come from $\theta_{1}=-20^{\circ }$, $\theta_{2}=0^{\circ }$, $\theta_{3}=8^{\circ }$. SNR = 10 dB. In both cases, two different numbers of snapshots are considered: *L* = 100 and *L* = 5. The spatial spectrum search is performed over $\left[-90^{\circ },90^{\circ }\right]$ with step size $0.01^{\circ }$. The angles corresponding to the top two highest peak positions in the spectrum allow estimation of the DOAs of incoming signals. Figure 1 and Figure 2 show the PSD of the methods with two and three coherent sources, respectively. The vertical dashed lines indicate the true values of DOAs.

As shown in the two-source case in Figure 1, FBLP-SVR and FBLP perform better with 100 snapshots than with 5 snapshots in terms of the positions of the peaks and stability of curves across the spectrum. When there are 100 snapshots, these two methods are able to detect the true DOAs. The PSD curves of FBLP and FBLP-SVR are similar to each other. However, in the scenario where there are only five snapshots, the FBLP-SVR method can accurately detect the signals while the standard FBLP fails. Similar results can be observed in a three-source case, as shown in Figure 2. The curves of FBLP-SVR have no false peaks, even when the number of snapshots is small.

### 4.2. Performance versus Angle Separation

**Figure 3:**
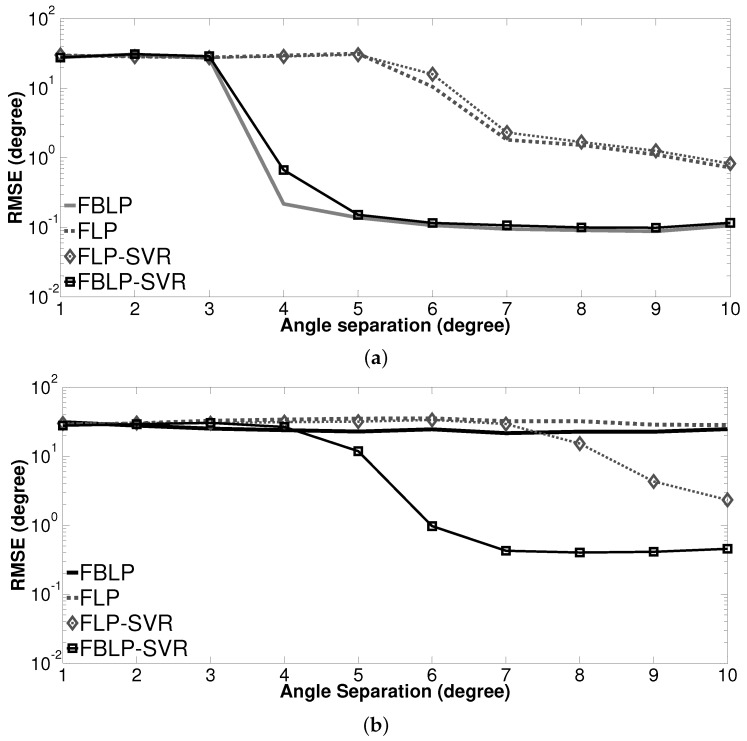
Root mean square error (RMSE) of direction-of-arrival (DOA) estimation as a function of angle separation. (**a**) number of snapshots = 100; (**b**) number of snapshots = 5.

In the second simulation, the statistical performance of the proposed FBLP-SVR versus the angle separation between the incoming signals is assessed. For comparison, we also collect the results of the standard FBLP, FLP, and SVR-based FLP method (FLP-SVR). There are two sources in the performance analysis. One source is fixed at direction $\theta_{1}=0^{\circ }$, while the other comes from $\theta_{2}=\theta_{1}+∆\theta$ with the same power. SNR = 15 dB and ∆θ varies from $1^{\circ }$ to $10^{\circ }$. In the evaluation, root mean square error (RMSE) is applied based on 500 independent realizations. The definition of RMSE is given by [8]:

(12) $$RMSE=\sqrt{\frac{1}{KJ}\sum_{j=1}^{J}\sum_{k=1}^{K}{\left({\hat{\theta }}_{kj}-\theta_{k}\right)}^{2}}$$

where ${\hat{\theta }}_{kj}$ is the estimate of $\theta_{k}$ at the *j*th independent trial. *J* is the total number of trials. Figure 3 depicts the RMSE of DOA estimation versus angle separation for both 5 and 100 snapshots. As can be seen from Figure 3, all methods fail to detect the DOAs when the angle separation is small. When there are 100 snapshots and the angle separation is larger than $3^{\circ }$, the results of SVR methods (FBLP-SVR, FLP-SVR) are similar to the standard LP methods (FBLP, FLP); the RMSE of FBLP-based methods (FBLP, FBLP-SVR) is smaller than that of FLP methods (FLP-SVR, FLP) for every angle separation. When there are 5 snapshots, the standard FBLP and FLP cannot work, regardless of the angle separation. The performance of FBLP-SVR and FLP-SVR with five snapshots is inferior to that with 100 snapshots. FBLP-SVR has the best accuracy and outperforms FBLP, FLP, and FLP-SVR at low angle separations.

### 4.3. Performance versus Number of Snapshots

**Figure 4:**
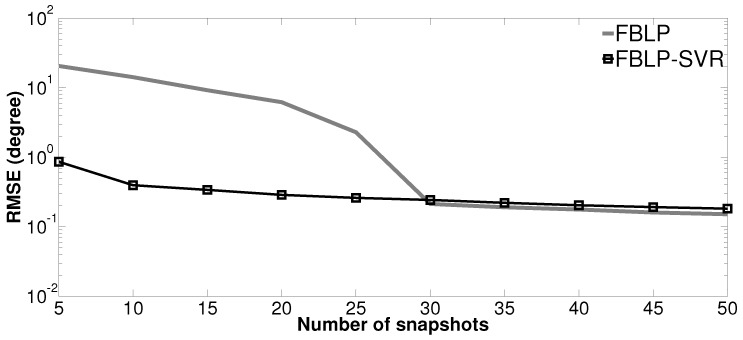
RMSE of DOA estimation versus number of snapshots.

In the third simulation, we test the performance of the proposed method as a function of the number of snapshots. The simulation conditions are similar to the second simulation, except the two coherent sources are from $\theta_{1}=0^{\circ }$ and $\theta_{2}=6^{\circ }$. The comparison is conducted between the standard FBLP and the proposed FBLP-SVR. The number of snapshots *L* varies from [5,10,15,20,⋯,50]. At each number of snapshots, the statistical analysis is conducted with 500 independent trials. Figure 4 shows the RMSE of DOA estimation versus the number of snapshots. The RMSE of FBLP-SVR and FBLP decreases with the increase of the number of snapshots. When the number of snapshots is small, the proposed FBLP-SVR gives much better estimation result than the standard FBLP. As *L* gets larger, FBLP and FBLP-SVR achieve similar results.

In order to get an idea about the computational burden, the execution time is evaluated with L=5 during 500 simulations. The average time for a single simulation using the proposed FBLP-SVR is 0.2728 s, while the corresponding time for the standard FBLP is 0.2595 s with a computer equipped with a processor unit (CPU) of 2.7 GHz and 16 GB of RAM. This indicates that the integration of SVR with FBLP can greatly improve the estimation performance with only a small additional computational burden, especially when the number of observation snapshots is limited.

### 4.4. Performance versus SNR

**Figure 5:**
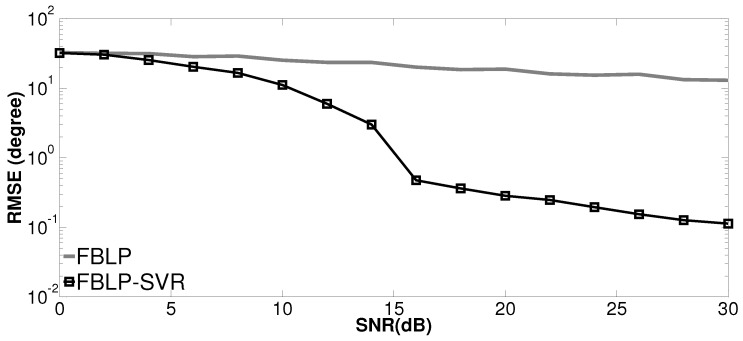
RMSE of DOA estimation versus signal-to-noise ratio (SNR) via 5 snapshots.

In the last simulation, we evaluate the performance of the proposed method with respect to SNR in coherent scenarios. The two sources come from directions $\theta_{1}=0^{\circ }$ and $\theta_{2}=6^{\circ }$, respectively. Only five snapshots are considered, and SNR varies from 0 to 30 dB. The RMSE is calculated with 500 independent trials for each SNR. The RMSE of angle estimation against SNR with 5 snapshots is plotted in Figure 5. It can be seen that FBLP-SVR has a more significant decrease of RMSE when SNR increases, compared with the standard FBLP.

## 5. Conclusions

This paper proposes a combined version of SVR and FBLP in the estimation of the DOAs of coherent incoming signals, taking advantage of their inherent features. The proposed FBLP-SVR allows to directly deal with coherent signals, and remains applicable with a limited number of snapshots. Simulation results prove the stability and robustness of FBLP-SVR with coherent signals and low snapshots, in comparison with FLP, FLP-SVR, and FBLP.

## Abbreviations

The following abbreviations are used in this manuscript:

DOA	Direction-of-Arrival
FBLP	Forward-Backward Linear Prediction
SVR	Support Vector Regression
SNR	Signal-to-noise
SS	Spatial smoothing
FB	Forward–backward
LP	Linear prediction
AR	Auto-regressive
ARMA	Auto-regressive moving average
FLP	Forward linear prediction
BLP	Backward linear prediction
MUSIC	Multiple Signal Classification
ESPRIT	Estimation of Signal Parameters via Rational Invariance Technique
ULA	Uniform Linear Array
AGWN	Additive Gaussian white noise
PSD	Power Spectrum Density
QP	Quadratic programming
RMSE	Root Mean Square Error
